# The Social Life of Infants in the Context of Infectious Disease Transmission; Social Contacts and Mixing Patterns of the Very Young

**DOI:** 10.1371/journal.pone.0076180

**Published:** 2013-10-16

**Authors:** Albert Jan van Hoek, Nick Andrews, Helen Campbell, Gayatri Amirthalingam, W. John Edmunds, Elizabeth Miller

**Affiliations:** 1 Immunisation, Hepatitis and Blood Safety Department, Public Health England, London, England; 2 Department of infectious disease epidemiology, London School of Hygiene and Tropical Medicine, London, England; Melbourne School of Population Health, Australia

## Abstract

Insight into how humans interact helps further understanding of the transmission of infectious diseases. For diseases such as pertussis, infants are at particular risk for severe outcomes. To understand the contact pattern of infants, especially those too young to be vaccinated, we sent contact diaries to a representative sample of 1000 mothers in the United Kingdom. We received 115 responses with a total of 758 recorded contacts. The average number of daily contacts for an infant was 6.68 overall and 5.7 for those aged ≤10 weeks. Of the latter, 2.1 (37%) contacts were with non-household members and were >15 minutes duration, suggesting that a cocooning programme may miss a substantial proportion of exposures leading to disease transmission. The least contact was between adolescents and infants. Thus the impact of adolescent (pertussis) vaccination on infants would likely be limited, unless it reduces transmission to other age groups whose contact with infants is greater.

## Introduction

A great number of infectious diseases are solely transmitted directly from person to person, hence an understanding of contact patterns between individuals is essential in understanding the transmission of these diseases [1]–[3]. Despite a dominant role of contact patterns in the transmission process it is a relatively under studied field.

When documenting contact patterns it is important not only to have a qualitative description of the type and nature of contact but also a quantitative description; the average number of contacts between gender/age groups/risk group on a day. This quantitative description (contact matrix) is a key input in infectious disease models that simulate the transmission of diseases in the population [3].

Several age-stratified contact studies have been performed [3]–[10] yet the contact patterns of very young infants remain poorly described. This is despite the fact that for certain infections, such as pertussis, the greatest risk of a severe outcome is shortly after birth, before the infant becomes eligible for vaccination. Under these circumstances indirect protection of the young infant by reducing its exposure to infectious individuals, either by vaccinating close contacts or inducing herd immunity at the population level, may be needed [11]. Predicting the impact of such a strategy requires detailed information on the contact patterns of very young infants. Such interventions remain topical with an outbreak of pertussis in England (end 2011–2012) resulting in 14 reported infant deaths in 2012 [12].

We performed a contact study among a sample of mothers with infants under 12 months of age, with a focus on those aged up to 10 weeks, in order to define more precisely the contact matrix for infectious disease transmission models. We measured the number of contacts between infants and others, defining both the duration of the contact-event and whether there was a skin-to-skin interaction - two key dimensions that affect the transmission risk.

## Methods

### Questionnaire

Contact information was collected by asking the mother/guardian to record all the contacts on one day that met the following definition: an interaction in close proximity with three or more words directed to the infant or a physical skin-to-skin contact between infant and another person.

A background questionnaire and a contact diary were developed in line with previously conducted contact surveys [3], [4].

The background questionnaire collected information on age, gender, number of siblings, ethnic group of the infant, the highest level of education among household members and family composition including age, sex and relation to the infant.

The contact diary collected information on the persons who contacted the infant; namely gender, age (or age range), whether the person was a member of the household, location of the contact (home, nursery/childcare, work, transport, leisure or other), the total time spent in presence of the infant (<5 min, 5–14 min,15–59 min,1–4 hours, more than 4 hours), if the person touched the infant, if this contact lasted longer than 5 minutes and how often the infant usually would have contact with this person (daily, once/twice a week, once/twice a month, less than once per month, never met before). Also information was gathered on the maximum distance from home travelled by the infant on the survey day. A maximum of 50 contact events could be recorded on the questionnaire. Questionnaires were sent on 8–9 November 2012 by mail, and included a pre-stamped return envelope. Where there was no initial response, reminders were send on the 5^th^ of December 2012. To prevent parents picking a convenient day, with a low number of contacts, a specific day of the week was assigned (50% weekend days), parents were however free to pick the week.

### Sample

We identified mothers in collaboration with Bounty (www.bounty.com), a parenting club providing support to pregnant women and mothers. Over ninety-five percent of first time mothers in the UK as well as a smaller proportion of the multiparous mothers receive information packs from Bounty at one or different stages of pregnancy [13]. Bounty selected 1000 children aged 12 months or younger from their database by a constrained random sample among those who had given consent to be approached by third parties.

The sample was constrained as follows: 50% of the infants to be aged ≤10 weeks (due to the delay between selecting parents and the delivery of the questionnaires the first two weeks of life were not included), 50% born to multiparous women, 50% in socioeconomic grades A,B,C1 (middle class) as defined by the national readership survey [14] and 50% in C2,D,E (working class). Mothers were sampled from the whole of the United Kingdom (England, Wales, Scotland and Northern Ireland). See a specification of the sample in File S1.

### Ethical approval

Given that the study did not include patients and was purely observational, no formal ethical approval was needed. Formal waivers were obtained from the National Research Ethics Service and the Research Services Review Sub Group of Public Health England.

### Statistical analysis

The influence of co-factors on the total number of contacts on a day was elucidated by a negative binomial regression model, modelling the effect of; being ≥11 weeks of age, contacts recorded on a week day or weekend day, sex of the infant, total number of household members, ethnicity and socio-economic status. Differences in the nature of contacts (by contact type/location/duration/frequency) between those aged ≤10 weeks and ≥11 weeks was investigated by a random effects logistic regression model to take into account individual differences in the number of contacts. When the random effects model did not converge a Fisher exact test was applied, comparing the number of infants who had at least 1 type of these contacts to those with none.

The contacts are presented in two ways: the average number of contacts between an infant with age group *i*, and the average number of contacts of an average person in age group *i* with the infant. The latter was calculated as follows: the average number of contacts of the infant with age class *i* was multiplied by the total number of live births in the UK, subsequently this total number of contacts was divided by the UK population sizes of age group *i* to obtain the average number of contacts between an infant and an individual member of age group *i*. For consistency the age groups used are in line with previous publications; 5 year age groups until 70+ [3].

To correct for over or under sampling the contribution of participants to the overall outcome were reweighted. The weighting was the product of the theoretical distribution over socio-economic class (A = 4%, B = 22%, C1 = 29%, C2 = 21%, D = 15%, E = 8%), aged below (10/52) or above 10 weeks (42/52), weekday (5/7) or weekend (2/7) and having no siblings (53%; only based on married couples) or multiple (47%)[15]. The obtained product was divided by the sum of the weights of all included responders such that the total of all weights summed up to one. When there was a focus on a difference between age or weekday these probabilities were excluded in the weights.

Confidence intervals were obtained by bootstrap of the data (1000 samples). In case two different samples were compared (for example week/weekend) the weights for each sample summed to one in each iteration. The population size for the UK was obtained from the Office of National Statistics [16]. All analyses were performed with R 2.15.0 [17].

## Results

### Response

The responders were equally distributed over the groups in the random sample (table 1). A total of 115 mothers returned the contact diary and 25 remained undelivered resulting in an overall response rate of 12%. There was a slightly higher response among the more affluent and the proportion of ethnic whites was slightly higher in our sample compared to the proportion in the infant population in 2010 (population 74%; sample 84%) [16]. The majority of survey infants (96/115; 83%) had a family structure of a father, mother and/or sibling (7 had also other family members in the household), see table S2 in File S1.

**Table 1:** Characteristics of the participating infants.

Total questionnaires sent	1000
Response	115
First child in the family	62 (54%)
Social grade A,B,C1 (population norm: 55%)	77 (67%)
Aged 10 weeks or younger	62 (54%)
Male infant	62 (54%)
Recorded contacts for a week day	61 (53%)
Ethnicity: white (population norm: 74%)	98 (85%)

### The total number of contacts per day

**Figure 1:**
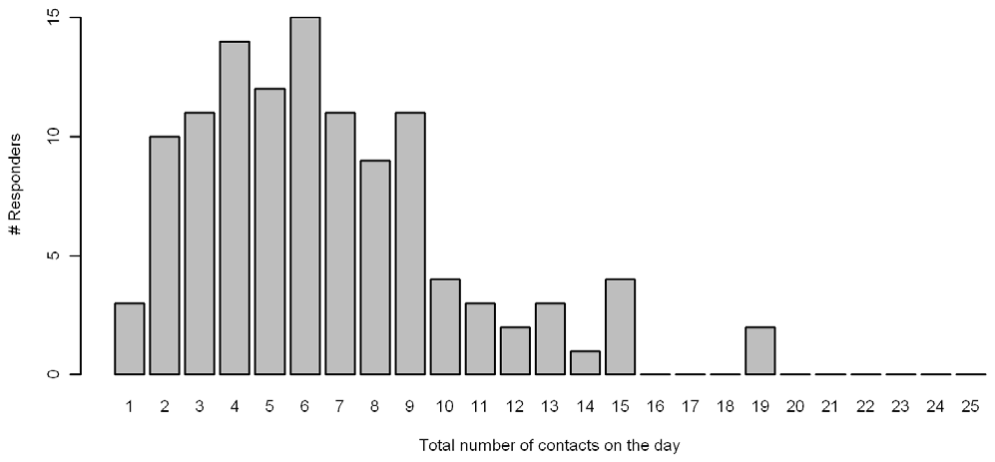
Histogram of the total number of contacts per day/per respondent.

Among the 115 study infants a total of 758 contacts were recorded, the minimum was 1 contact, and the maximum 19, see figure 1 (the maximum of 50 contacts per day was therefore never reached). The age of the contacts ranged from 0 to 92 years, with only 4 recorded contacts between under one year olds. The average number (reweighted) of contacts of an infant was 6.68 contacts per day. However 37 (32%) participants did not record the age of (some) of their contacts. These participants were excluded from the analysis of the contacts by age, affecting 317 contacts (41% of total), but were included in the analysis of the total number of contacts over the course of a day.

In the negative binomial regression including age (≤10 weeks, ≥11 weeks), gender, ethnicity, socio-economic status (ABC1 and C2DE) and number of household members as explanatory variables for the total number of contacts, revealed that aged ≥11 weeks and an increased number of household members significantly increase the number of contacts (p<0.01). Although not significant; infant contacts recorded at the weekend are higher than those recorded on a week day (p = 0.07). See table 2.

**Table 2:** Outcome of the negative binomial regression for the number of contacts per infant.

	Mean # contacts (min-max)	RR	95% CI	P-value
*Age of infant*				
≤10 weeks	6 (1–19)	1		
≥11 weeks	7.3 (1–19)	1.46	1.2–1.79	<0.01
*Day of recording contacts*				
Week day	6.2 (1–19)	1		
Weekend day	7.1 (2–19)	1.19	0.98–1.44	0.08
*Household size*				
1 or 2	5 (1–13)	1	NA	NA
3	5.8 (2–13)	1.20	0.83–1.2	0.33
4	6.9 (3–15)	1.36	0.94–1.36	0.10
>4	9.1 (4–19)	2.19	1.44–3.33	<0.01

### Difference between those aged ≤10 weeks and ≥11 weeks

There are some significant differences in the overall contacts between those aged 10 weeks and under and those over 10 weeks; younger infants had more contact with household members, more contacts that lasted longer than 4 hours, more contacts recorded at home and more daily contacts, see table 3. The median distance travelled from home by an infant was 3 miles, and was similar between infants aged up to 10 weeks and those over 10 weeks (see also table S3 in File S1).

**Table 3:** Characteristics of the contact-events split by whether the study infant was ≤10 weeks or 11 weeks-12 months old.

	≤10 weeks		≥11 weeks–12 months		P-value Random effects logistic regression
	Survey response	Weighted response	Survey response	Weighted response	
*Study infants*	*62*		*53*		
Total contacts	372		386		
Contact per respondent (unweighted and not by age)	6.0	5.7	7.3	7	
Male contact	146 (38%)	35% (31%–40%)	158 (41%)	40% (32%–45%)	P = 0.25
Contact is a household member	180 (47%)	48% (42%–55%)	130 (34%)	34% (29%–40%)	P<0.01
Location of contact:					
Home	254 (66%)	48% (42%–55%)	188 (49%)	34% (29%–40%)	P<0.01
Nursery or childcare	6 (2%)	2% (0%–4%)	25 (6%)	8% (3%–15%)	P = 0.8
Work	12 (3%)	5% (0%–13%)	0 (0%)	0% (0%–0%)	P = 0.13*
Transport	24 (6%)	6% (3%–10%)	43 (11%)	12% (6%–18%)	P = 0.24
Leisure (Shopping, swimming park etc.)	89 (23%)	24% (14%–35%)	107 (28%)	24% (16%–34%)	P = 0.57
Other (GP visit, mother/infant group etc.)	72 (19%)	18% (9%–28%)	114 (30%)	27% (16%–38%)	P = 0.71*
Duration of contact:					
<5 minutes	38 (10%)	10% (5%–14%)	50 (13%)	14% (8%–20%)	P = 0.34
5–14 minutes	34 (9%)	10% (5%–17%)	27 (7%)	8% (3%–14%)	P = 0.73
15–59 minutes	51 (13%)	13% (8%–18%)	55 (14%)	17% (10%–25%)	P = 0.76
1–4 hours	92 (24%)	24% (17%–33%)	120 (31%)	27% (19%–35%)	P = 0.16
More than 4 hours	168 (44%)	42% (36%–49%)	129 (34%)	33% (26%–42%)	P = 0.02
Physical contact	314 (83%)	80% (72%–87%)	296 (77%)	75% (68%–82%)	P = 0.02
Physical contact longer than 5 minutes	228 (59%)	58% (51%–67%)	194 (50%)	48% (41%–56%)	P = 0.02
Frequency of contact:					
Daily	203 (53%)	52% (45%–60%)	150 (39%)	41% (35%–47%)	P<0.01
Once or twice a week	69 (18%)	19% (13%–26%)	101 (26%)	28% (21%–36%)	P = 0.04
Once or twice a month	32 (8%)	8% (5%–12%)	53 (14%)	12% (8%–17%)	P = 0.13
Less than once per month	50 (13%)	11% (5%–17%)	45 (12%)	10% (5%–17%)	P = 0.73
Never met before	30 (8%)	8% (5%–11%)	32 (8%)	7% (4%–10%)	P = 0.71

* P-value based on the Fisher exact test comparing the number of infants with at least 1contact of this type with those none.

**Figure 2:**
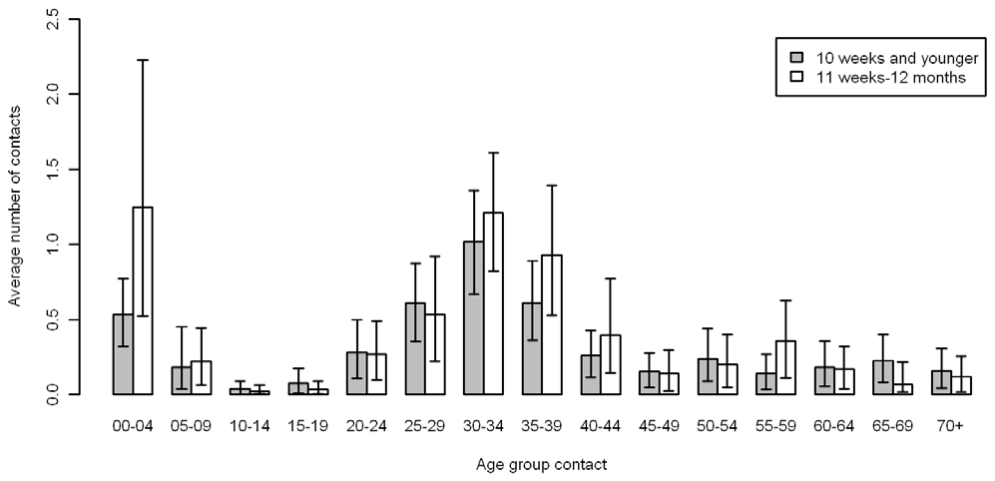
Average number of daily contacts of study infants aged ≤10 weeks and 11 weeks to <12 months with persons in age group *i*, using a weighted sample; confidence intervals are obtained by bootstrap (excluded 38 respondents with missing age data).

There were minimal differences in the contact patterns of younger (≤10 weeks) and older infants with people in other age groups. In figure 2 and table S4 in File S1 the number of contacts of an infant with a person from a specific age group is shown. The mean number of contacts was between 0.025 (≥*11 weeks* with 10–14 year olds) and 1.28 (≥*11 weeks* with 0–4 year olds). The main difference between those aged 10 weeks or less and those older than 10 weeks was the amount of contact with children aged 0–4: the average daily number of contacts was 0.54 for those aged ≤10 weeks compared to 1.28 in older infants. When the number of contacts is defined as the average number of contacts of someone in age group *i* with an infant, see figure S1 and table S4 in file S1, the lowest rate was 0.002 contacts per day between someone aged 10–14 with an infant aged 10 weeks or younger, and the highest rate was for children aged 0–4 with an infant aged ≥*11 weeks* with 0.20 contacts per day.

### Gender difference

There were more contacts with females compared to males: 60% of the contacts were with females, see table 3. Infants had 29% (95% CI: 9%–49%) less contact with males compared to females. This difference widened when only persistent physical contacts are included to 36% (95% CI: 22%–49%), see also figure S2 in File S1. In figure S3 in File S1 the contacts are differentiated by gender and age, which shows that this gender difference holds over all age groups.

### Contacts with members of the household

There is a difference in the contacts differentiated by duration and intimacy level between members of the household and non-members (for clarity: all these contacts can occur at the home of the infant). For infants age ≤10 weeks 55% of contacts (an average of 3.1 per day) were with non-household members. However, this depends on the duration of the contact, so that 37% of contacts were with non-household members for duration of 15 minutes and longer and only 4% of contacts lasting over 4 hours were with non-household members. See table 4.

**Table 4:** The average number of contacts recorded for a study infant aged ≤10 weeks, differentiated by the duration (contact at least x minutes) and physical nature of the contact.

A) contacts with household members					
	All durations	Duration at least 5 minutes	Duration at least 15 minutes	Duration at least 1 hour	Duration at least 4 hours
No physical contact	0.04	0.03	0.02	0.02	0.02
Physical contact <5minutes	0.49	0.49	0.48	0.47	0.44
Physical contact >5 minutes	2.01	1.98	1.89	1.88	1.68
Total	2.55	2.51	2.4	2.37	2.14

B) contacts with non household members					
	All durations	Duration at least 5 minutes	Duration at least 15 minutes	Duration at least 1 hour	Duration at least 4 hours
No physical contact	1	0.67	0.53	0.41	0.03
Physical contact <5minutes	0.77	0.57	0.36	0.17	0.01
Physical contact >5 minutes	1.33	1.3	1.17	0.79	0.2
Total	3.11	2.55	2.07	1.37	0.25
Percentage of total contacts	55%	45%	37%	24%	4%

For A) contacts with household member and B) contacts with non household members. Both show weighted contacts.

Looking at all types of contacts infants had an average 24% (95% CI: −0.9%–46%) *fewer* contacts with household members compared to non-household members. However this changed when only persistent physical contacts are included, as there were 55% (95% CI: 13%–118%) *more* contacts with household members compared to non-household members (see also figure S4 in File S1).

Over 90% of all mothers, fathers and siblings had skin-to-skin contact with the infant on the sample day, see table 5. Only in the case of extended physical contact (>5 min) was there a significant difference between the mother and all other members of the household.

**Table 5:** The household based contacts of the study infant, split by the nature of contact and by mother, father, other siblings and other family members.

Family member	Conversational	Physical	Physical >5 min	Total number of infants with family member
Mother	97% (101)	96% (100)	92% (96)	104
Father	92% (90)	91% (98)	82% (80)*	98
Siblings	94% (65)	93% (64)	45% (31)*	69
Other family	63% (12)*	58% (11)*	47% (9)*	19

Percentage marked with a star are significant less compared to the mother (Chi-square p<0.05).

### Weekend vs weekday

There was almost no difference in the age structure of contacts on weekends and on weekdays, apart from more contacts with 5–9 year olds during the weekend (see figure S5 in File S1).

## Discussion

To our knowledge this is the first study that focuses on the contact pattern of infants in their first year of life, with specific attention on those too young to be fully vaccinated. The study included 115 participants who were in contact with 758 different individuals. In the context of infectious disease transmission not all of these contact-events are equally important, although this depends on the pathogen as the path of transmission differs. When the pathogen is transmitted by cough a conversational contact can be enough to spread the disease; however when the pathogen is transmitted by fluids or by skin-to-skin contact more intimate interaction may be needed.

Perhaps the most important observation in a public health perspective is that there was almost no contact between adolescents and those too young to be vaccinated. These results are of particular interest in the context of adolescent pertussis vaccination programmes, now being adopted by some countries to reduce disease burden among this age group and potentially to protect infants [18]. Due to the very low contact between these two age groups any reduction of disease among infants as a result of reduced exposure to infected adolescents is likely to be limited. Should adolescents play a dominant role in the overall disease transmission to age groups which do have frequent contact with infants then an adolescent vaccination strategy will have an indirect protective effect on disease in infants. However, in the case of pertussis vaccination, where the duration of protection after a booster dose of (acellular) vaccine is likely to be less than after a natural booster [19], [20], a booster dose given in adolescence might delay disease to later in life. This could increase disease incidence among young parents and increase transmission to their infants, leading to a counterproductive programme effect. Use of dynamic transmission models to explore such indirect effects before adoption of an adolescent booster programme could help clarify the conditions under which such a counterproductive effect would be generated.

The within household contacts are of interest in the context of household based interventions, as for example a cocooning strategy against pertussis, where one or more family members are vaccinated to prevent disease transmission. Such a programme is only successful if the vast majority of relevant contacts are within the household. For example, if a threshold of 15 minutes is used an estimated 37% of all the contacts are still with non-household members. Although the minimal duration of exposure to achieve transmission of pertussis is unknown, a 15 minutes threshold is indicated in post-exposure measles and varicella guidelines [21], [22], two diseases likely to be more (measles) or less (varicella) contagious compared to pertussis. This suggests that a cocooning programme may miss a substantial proportion of exposures likely to lead to disease transmission. More direct protection of the infant, by vaccinating the new born or the pregnant mother against pertussis, may therefore be needed for more complete protection. From these two options vaccinating the mother seems the best option as it induces direct as well as indirect protection. A recent household transmission study from the Netherlands suggests that the mother plays an even more important role in disease transmission within the household than is suggested by our contact study, as the mother also plays a key role in transmission to other household members who might infect the infant [23].

The contact pattern of infants is not-assortative. An important outcome of the previous contact study [3] is the assortativeness of mixing; people mix most with people of the same age groups. Infants differ from this. In this study there were only 4 recorded contact-events with other under ones showing that infants only interact with people who are older than themselves. The disease transmission between infants will therefore be minimal.

The average number of contacts of an infant is lower than the average number of contacts measured in the age group 0–4 years in the previous contact survey in Great Britain, 6.68 (reweighted) versus 8.8 [3]. Although the proportion of contacts including skin-to-skin contacts is similar among infants compared to the 0–4 years old (both 76%) the total absolute average number of skin-to-skin contacts is lower (5.1 vs 6.7) [3]. Therefore an infant can be considered less exposed compared to other children. However, in our analysis we focussed on contact-types relevant for aerosol and touch-based transmission; for other infections blood-to-blood or the faecal-oral route are applicable. For these routes there might be important differences in exposure between those under and those over 1 year of age.

An interesting observation in our study is the higher number of contact of the infants with females compared to males. This elevated level of contact is seen for all age groups and the difference between the sexes becomes stronger using only the longer physical contacts. Fathers had significantly less sustained physical contact with the infant compared to the mother.

There are a number of caveats in this study. The response rate was not high, even with a reminder being sent out. However the sample was adequately distributed over the constrained sample and the final sample was of sufficient size to observe significant differences between groups. There were only 15 infants included in the previous contact study, therefore this study adds markedly to previously published data [3]. To counterbalance obvious selection biases and over and under sampling we have reweighted our results. However there are numerous potential unobserved biases which were not accounted for; biases such as a potential difference in response between urban and rural participants and a lower response among people with a day job. Future studies can experiment with other collection methods or improve recruitment to achieve larger sample sizes and reduce the influence of these potential biases.

The people who did not fill in an age estimate for some of their contacts had a higher number of contacts per respondent compared to the group who did fill in this information. Therefore the contacts-structure by age was slightly biased towards the group of respondents with a lower number of contacts.

There is a potential bias towards infants not attending nursery on the day the diary should be completed. The data however seems in line with expectations, as the number of infants who were observed having contact at a nursery was in line with the population estimate for under 2 s in England, 15% (9/61) vs 19% (254,800/1,349,000) [24], [25] (p = 0.47 Chi –square test).

As the study is dependent on participation and parents observing and recording contacts (and the age of contacts) there is likely to be participation and observation/recall bias, especially those with a lot of short contacts who might have failed to remember these or the age of contacts [26].

Despite these potential limitations we believe that our study provides unique insight into the contact patterns of infants and provides essential quantitative data for evaluating the potential impact of different control strategies to protect this vulnerable group from exposure to serious infections.

## Supporting Information

File S1 Contains: Figure S1 Average number of daily contacts between someone in age group *i* with an infant aged below or above 10 weeks. As there are more children aged between 10 weeks and 12 months, an average person has more contacts with an average person in this age group, compared to those younger than 10 weeks. Figure S2 The number of contacts with males and females including all contacts, and only physical contacts. Figure S3 Average number of daily contacts of an infant with a male or female of age group *i*, using a weighted sample; confidence intervals are obtained by bootstrap (excluded 38 respondents with missing age data). Figure S4 The number of contacts within and outside the household including all contacts, and only physical contacts. Figure S5 Average number of daily contacts of an infant by weekend and week day, using a weighted sample; confidence intervals are obtained by bootstrap (excluded 38 respondents with missing age data)
(DOCX)
