# Supplementary material for: The Social Life of Infants in the Context of Infectious Disease Transmission; Social Contacts and Mixing Patterns of the Very Young
Source: PLoS One. 2013 Oct 16;8(10):e76180. doi: 10.1371/journal.pone.0076180 (PMC3797797; doi:10.1371/journal.pone.0076180)

**Supplementary material**

Table S1 Distribution of the sample by age of the infant, first time/multiple mum and socio-economic grade.

| Two groups | 2-10 weeks |  | 11 weeks-first year | |
| --- | --- | --- | --- | --- |
| Birthday between | 05/11/2012 |  | 10/09/2012 |  |
| And | 10/09/2012 |  | 20/11/2011 |  |
|  |  |  |  |  |
|  | 500 letters |  | 500 letters |  |
|  | First time mum | Multiple mum | First time mum | Multiple mum |
|  | 250 | 250 | 250 | 250 |
| A - upper middle class | 125 | 125 | 125 | 125 |
| B - middle class | week 63 | week 62 | week 63 | week 62 |
| C1 - lower middle class | weekend 62 | weekend 63 | weekend 62 | weekend 63 |
| C2 - skilled working class | 125 | 125 | 125 | 125 |
| D - working class | week 62 | week 63 | week 62 | week 63 |
| E - lowest | weekend 63 | weekend 62 | weekend 63 | weekend 62 |

Table S2 Family structure in the sample

| Family structure | # | Family structure | # |
| --- | --- | --- | --- |
| Unknown | 9 | Single father + at least one sibling | 2 |
| Single mum | 4 | Father + Mother | 43 |
| Mum + Other family | 2 | Father + Mother + at least one sibling | 46 |
| Single mum + at least one sibling | 1 | Father + Mother + Other family | 6 |
| Mum + Other family + at least one sibling | 1 | Father + Mother + as least one sibling + Other family | 1 |

Table S3 Travel distance of the participant

| Travel distance | n | % | % assuming UK are 0 | N | % | % assuming UK are 0 |
| --- | --- | --- | --- | --- | --- | --- |
| 0-1 mile | 10 | 20% | 16% (+19% =35%) | 12 | 29% | 23% (21%=43%) |
| 2-4 miles | 24 | 48% | 39% | 12 | 29% | 23% |
| 5-14 miles | 12 | 24% | 19% | 10 | 24% | 19% |
| 15+ miles | 4 | 8% | 6.5% | 8 | 19% | 15% |
| Unknown | 12 | NA | 19% | 11 | NA | 21% |

Figure S1 Average number of daily contacts between someone in age group *i* with an infant aged below or above 10 weeks. As there are more children aged between 10 weeks and 12 months, an average person has more contacts with an average person in this age group, compared to those younger than 10 weeks.


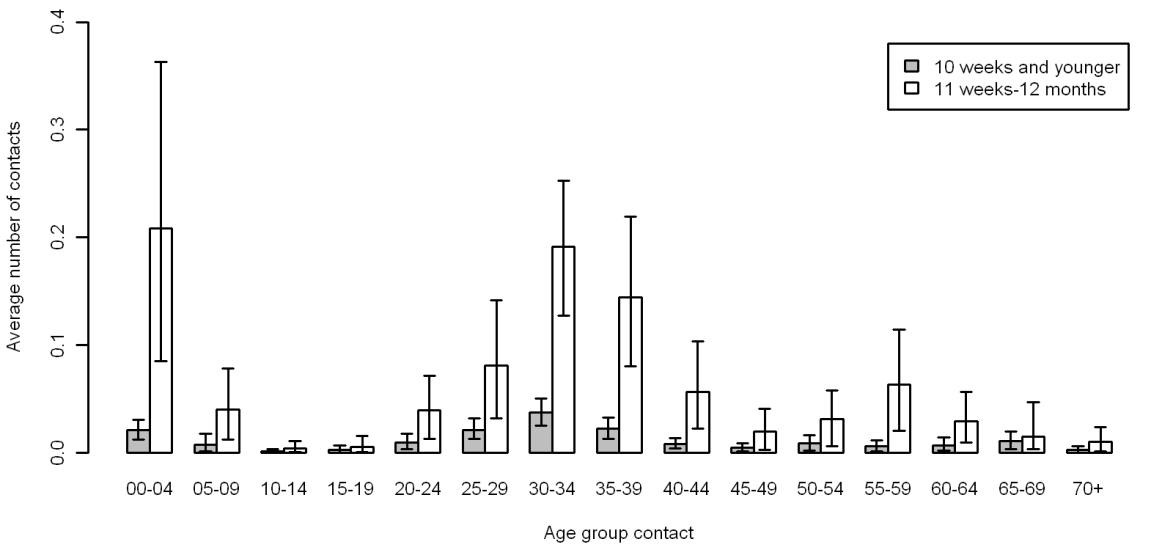


Table S4 The average number of contacts between infants aged 10 weeks and younger and 11 weeks and older with contacts of different age groups.

|  | Daily contacts of infants with those aged *i* | | Daily number of contacts of those aged *i* with the infant | |
| --- | --- | --- | --- | --- |
| Age of contact | 10 weeks and younger | 11 weeks-12 months | 10 weeks and younger | 11 weeks-12 months |
| 00-04 | 0.535 | 1.249 | 0.021 | 0.204 |
| 05-09 | 0.185 | 0.225 | 0.008 | 0.041 |
| 10-14 | 0.04 | 0.025 | 0.002 | 0.004 |
| 15-19 | 0.079 | 0.037 | 0.003 | 0.006 |
| 20-24 | 0.284 | 0.272 | 0.01 | 0.041 |
| 25-29 | 0.61 | 0.532 | 0.022 | 0.079 |
| 30-34 | 1.017 | 1.211 | 0.038 | 0.188 |
| 35-39 | 0.61 | 0.93 | 0.023 | 0.144 |
| 40-44 | 0.263 | 0.397 | 0.009 | 0.055 |
| 45-49 | 0.153 | 0.142 | 0.005 | 0.02 |
| 50-54 | 0.242 | 0.204 | 0.009 | 0.032 |
| 55-59 | 0.142 | 0.358 | 0.006 | 0.064 |
| 60-64 | 0.187 | 0.17 | 0.008 | 0.029 |
| 65-69 | 0.229 | 0.07 | 0.011 | 0.015 |
| 70+ | 0.158 | 0.122 | 0.003 | 0.011 |

Figure S2 The number of contacts with males and females including all contacts, and only physical contacts.


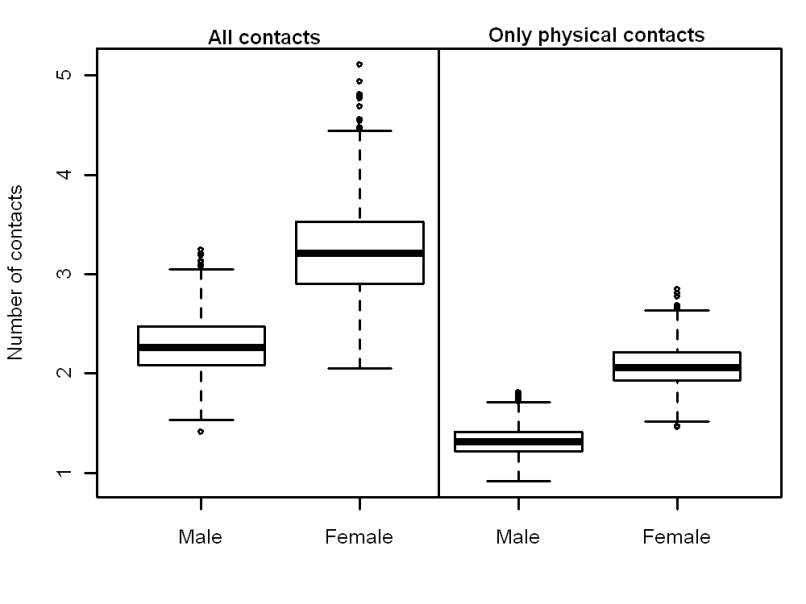


Figure S3 Average number of daily contacts of an infant with a male or female of age group *i*, using a weighted sample; confidence intervals are obtained by bootstrap (excluded 38 respondents with missing age data)


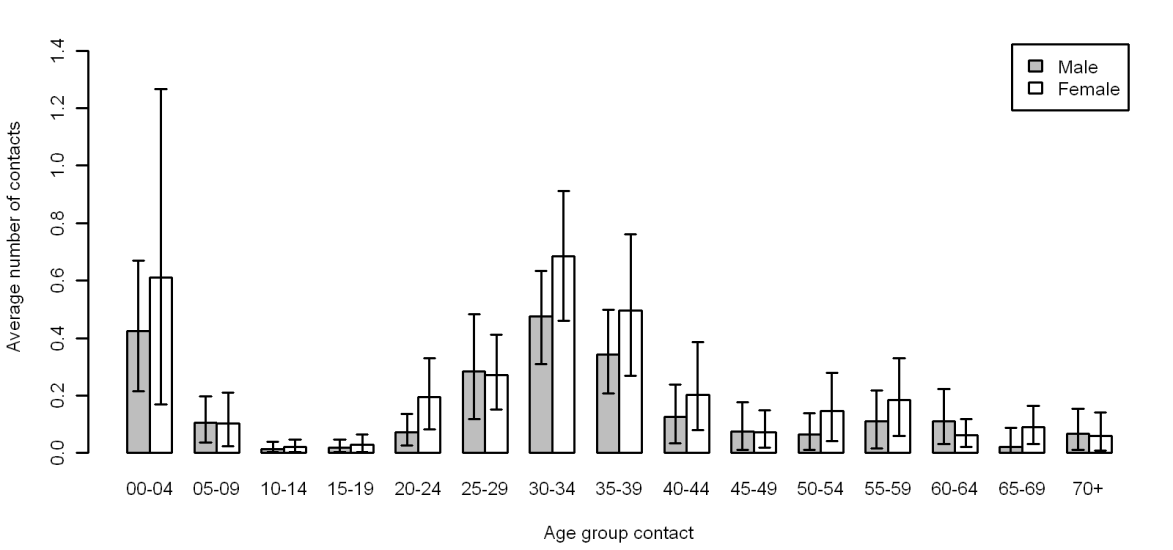


Figure S4 The number of contacts within and outside the household including all contacts, and only physical contacts.


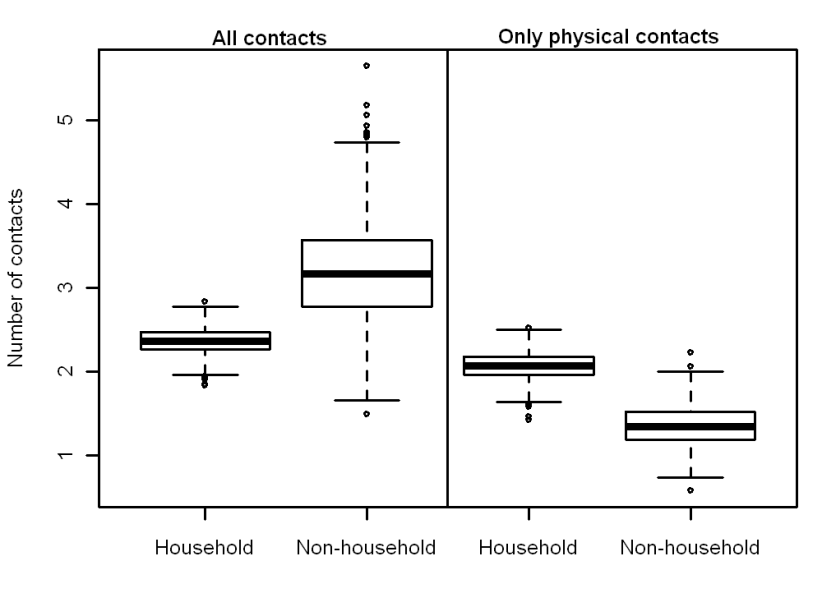


Figure S5 Average number of daily contacts of an infant by weekend and week day, using a weighted sample; confidence intervals are obtained by bootstrap (excluded 38 respondents with missing age data)


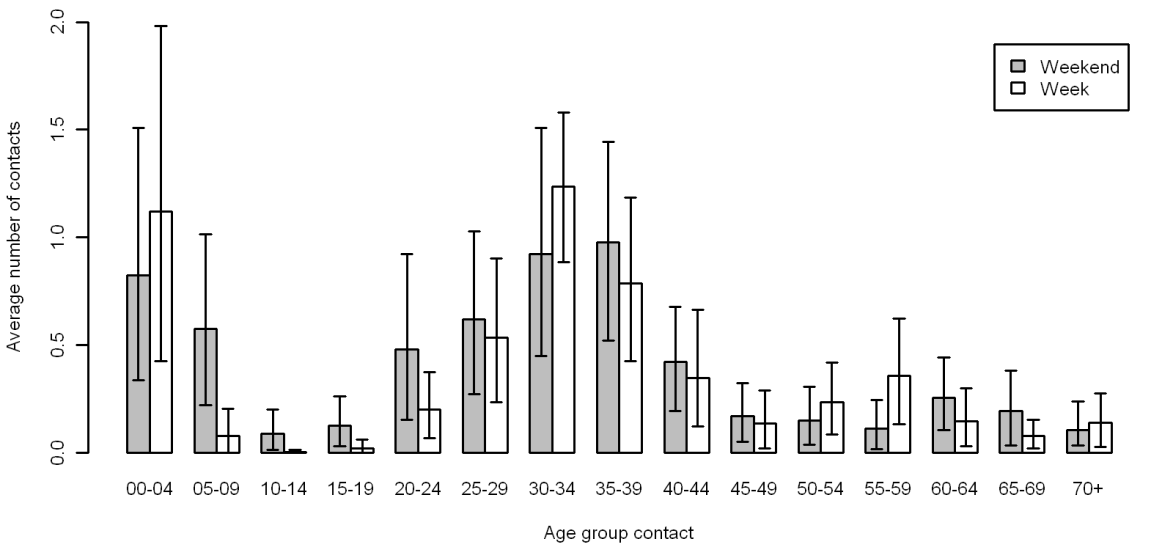

Supplement: File S1 — Contains: Figure S1 Average number of daily contacts between someone in age group i with an infant aged below or above 10 weeks. As there are more children aged between 10 weeks and 12 months, an average person has more contacts with an average person in this age group, compared to those younger than 10 weeks. Figure S2 The number of contacts with males and females including all contacts, and only physical contacts. Figure S3 Average number of daily contacts of an infant with a male or female of age group i, using a weighted sample; confidence intervals are obtained by bootstrap (excluded 38 respondents with missing age data). Figure S4 The number of contacts within and outside the household including all contacts, and only physical contacts. Figure S5 Average number of daily contacts of an infant by weekend and week day, using a weighted sample; confidence intervals are obtained by bootstrap (excluded 38 respondents with missing age data) (DOCX) [file pone.0076180.s001.docx]
